# Supplementary material for: Integrative and comparative single-cell analysis reveals transcriptomic difference between human tumefactive demyelinating lesion and glioma
Source: Commun Biol. 2022 Sep 9;5:941. doi: 10.1038/s42003-022-03900-0 (PMC9463163; doi:10.1038/s42003-022-03900-0)
Supplement: Supplementary file 2 — Supplementary information [file 42003_2022_3900_MOESM2_ESM.pdf]

Supplementary information

Table S1 Patient sample clinical information.

Diagnosis	Sex	Age	Symptom	Epilepsy	Location	Primary or Recurrence
Glioma	Male	28	Blunted response	No	Left frontal	Primary
Demyelinating lesion	Female	61	Headache	No	Right basal ganglia, bitemporal	Primary

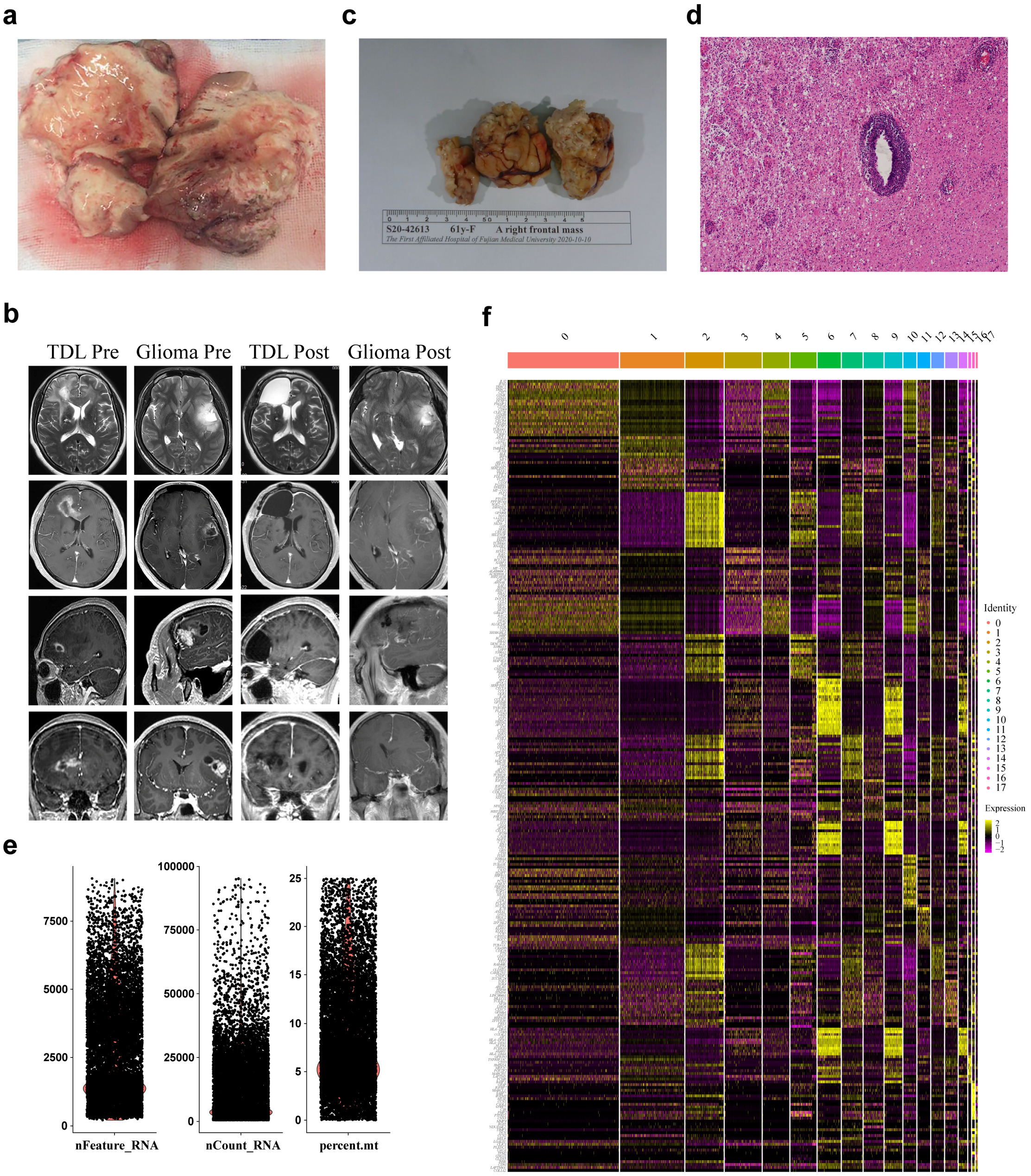

Figure S1 The radiological, pathological, and molecular information of TDL and glioma. **a** The lesion appearance of TDL after surgical excision. **b** Preoperative and postoperative radiological manifestation of TDL (first and third column) and glioma (second and fourth column). **c** TDL lesion for pathological examination. **d** Pathological results of TDL lesion. **e** The number of genes, unique molecular identifiers, and percent of mitochondrial genes in 11555 cells (from left to right). **f** Heatmap of top markers from each cluster. TDL, tumefactive demyelinating lesion; Pre. Preoperative; Post, postoperative.

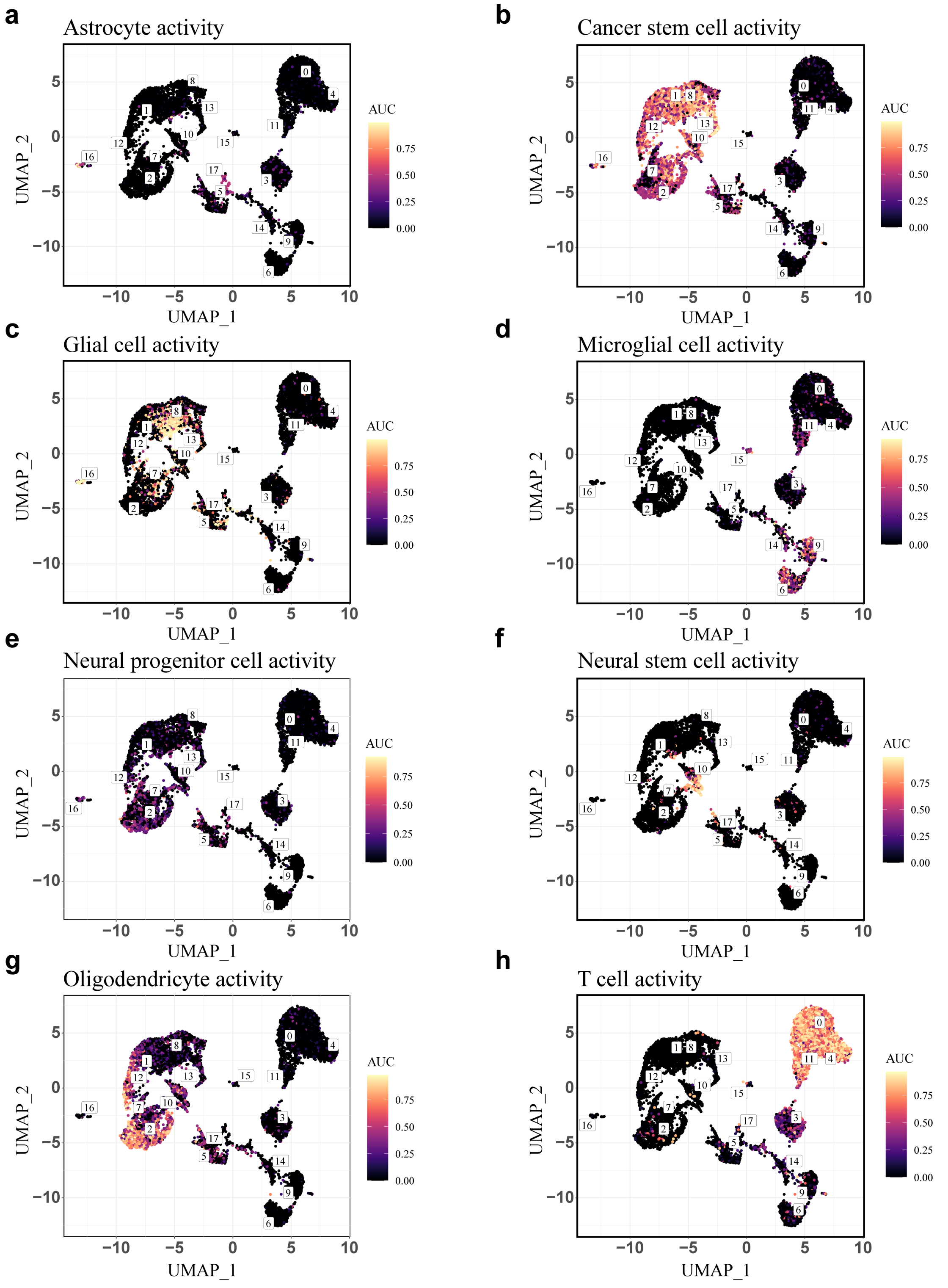

Figure S2 Individual cell AUC score overlay for selected canonical markers of 8 cell types. a Astrocyte. **b** Cancer stem cell. **c** Glial cell. **d** Microglial cell. **e** Neural progenitor cell. **f** Neural stem cell. **g** Oligodendricyte. **h** T cell.

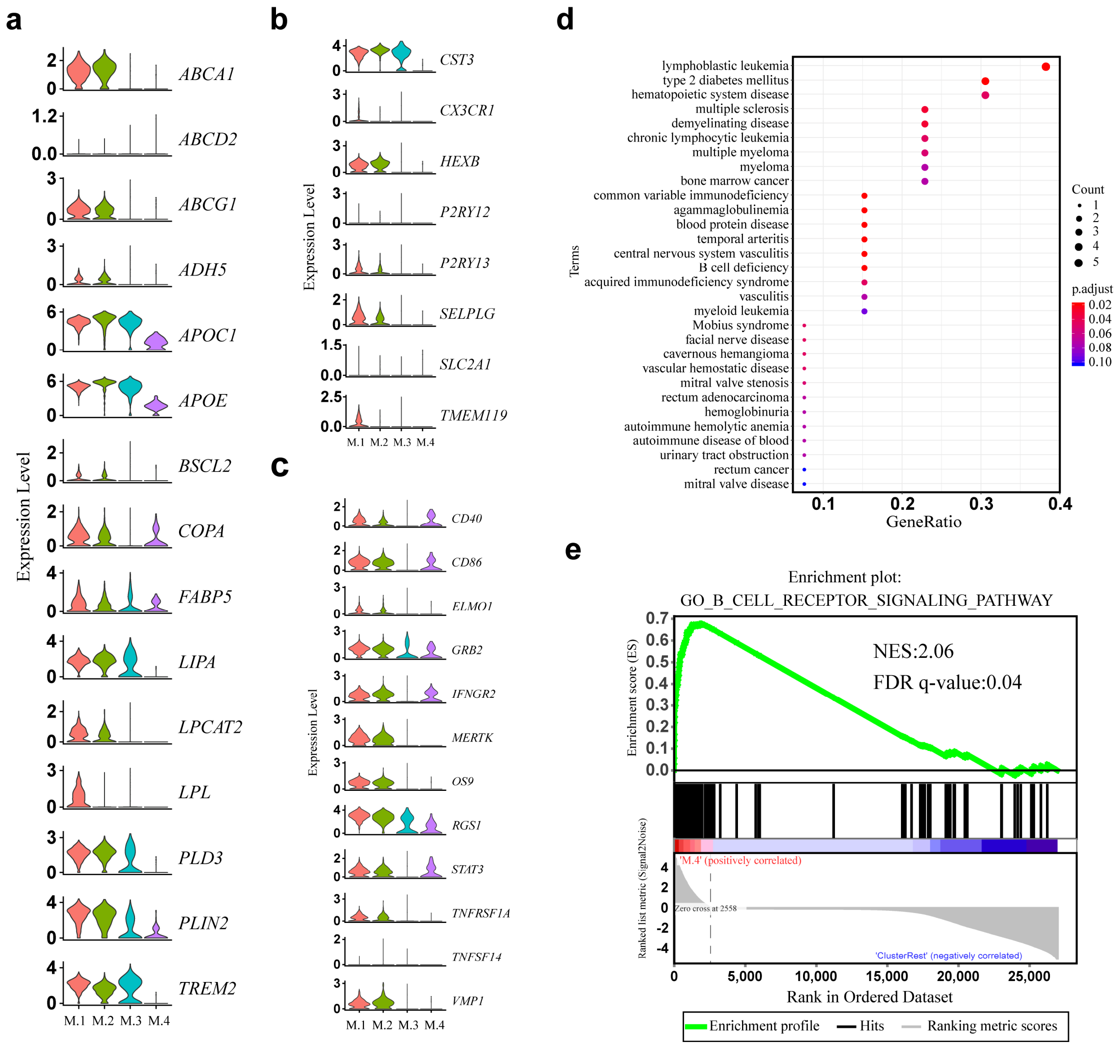

Figure S3 Expression of known markers and gene enrichment analysis. a Violin plot of activated microglial genes in 4 clusters. **b** Violin plot of homeostatic microglial genes in 4 clusters. **c** Violin plot of multiple sclerosis susceptibility genes in 4 clusters. **d** Bubble plot of disease enrichment analysis based on top 20 markers in M.4. **e** Gene set enrichment analysis for M.4. GO, Gene Ontology.

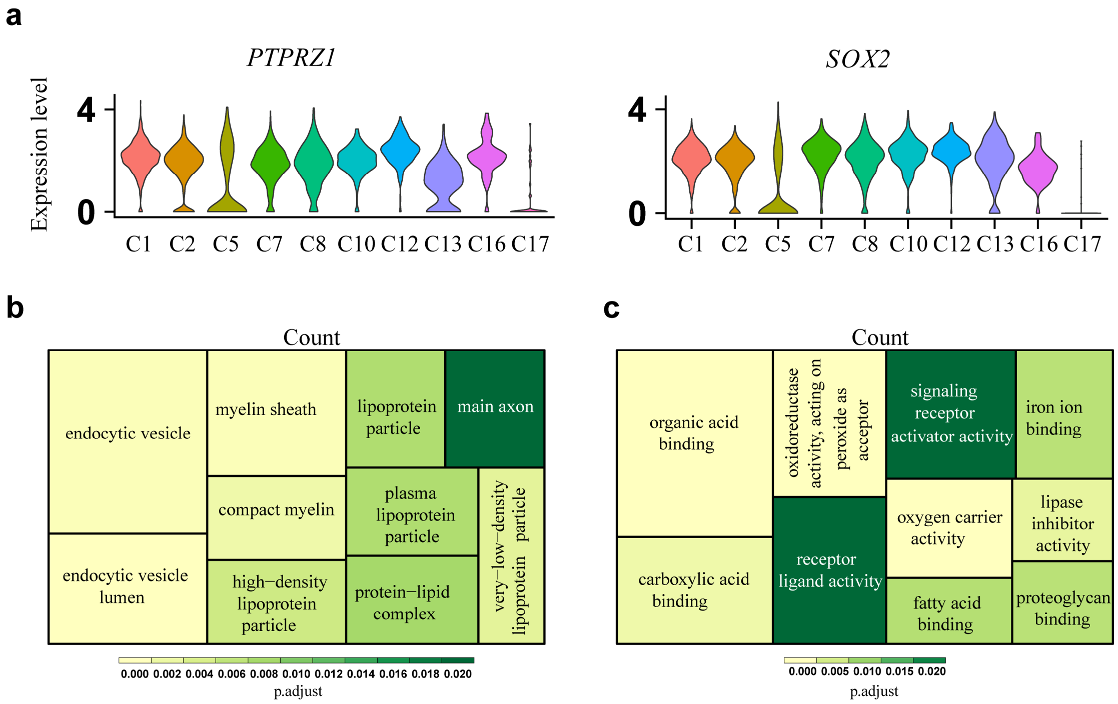

Figure S4 Expression of known markers and gene enrichment analysis. a Violin plot of glioma-related genes *SOX2* and *PTPRZ1* in non-immune cell clusters. **b** Treemap of Gene Ontology (GO) enrichment analysis for cellular component based on differentially expressed genes (DEGs) between C5 cells from TDL and glioma. **c** Treemap of GO enrichment analysis for molecular function based on DEGs between C5 cells from TDL and glioma. DEGs, differentially expressed genes; TDL, tumefactive demyelinating lesion.

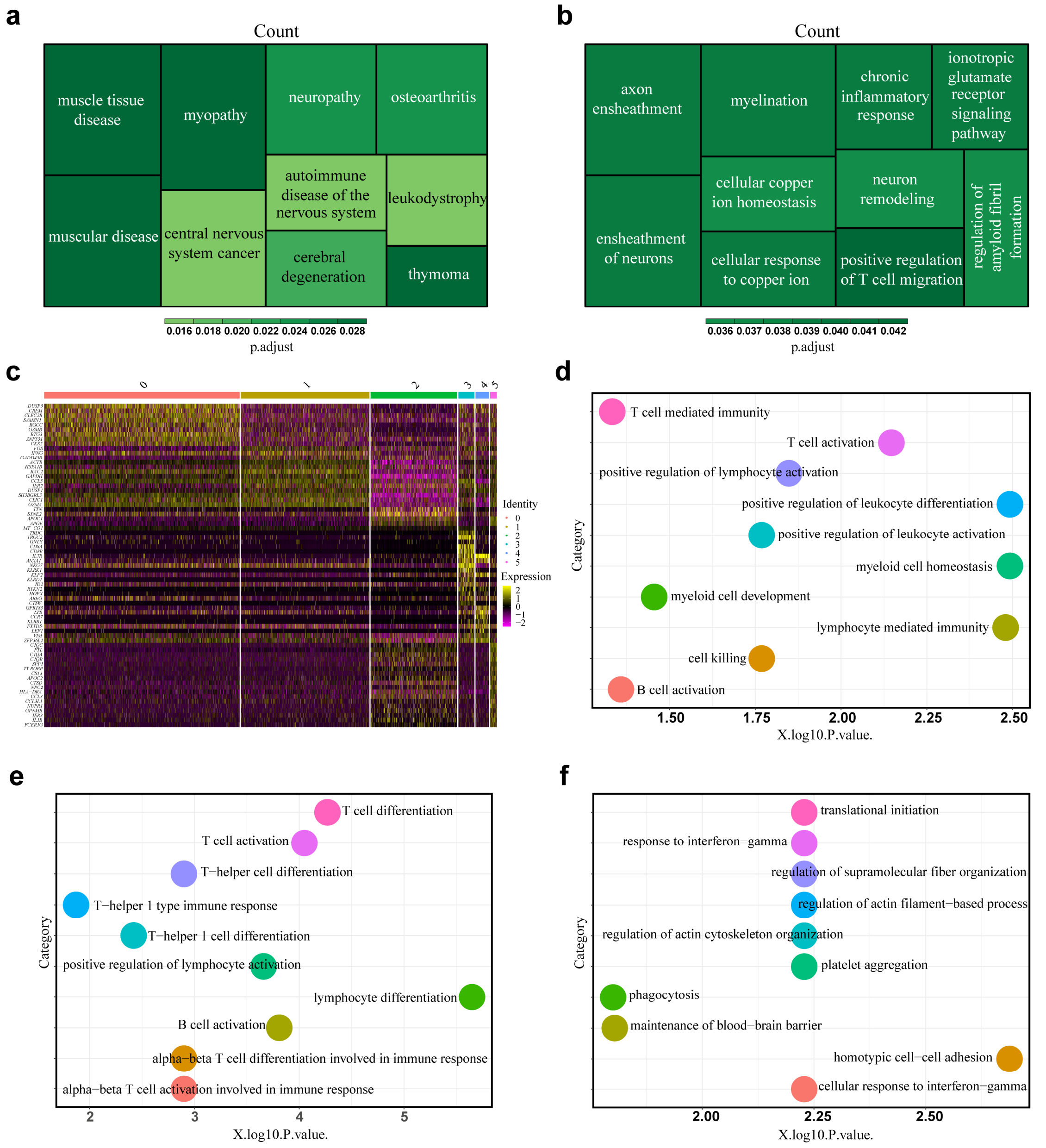

Figure S5 Gene enrichment analysis and expression of top markers in T cells. a Treemap of disease enrichment analysis based on differentially expressed genes (DEGs) between T cells from TDL and glioma. **b** Treemap of Gene Ontology (GO) enrichment analysis for biological process based on DEGs between T cells from TDL and glioma. **c** Top markers of T cell subclusters in TDL. **d** Dotplot of GO analysis (biological process) based on top 20 markers in C4. **e** Dotplot of GO analysis (biological process) based on top 20 markers in C3. **f** Dotplot of GO analysis (biological process) based on top 20 markers in C1. DEGs, differentially expressed genes; TDL, tumefactive demyelinating lesion; GO, Gene ontology.
