# Supplementary material for: Integrative and comparative single-cell analysis reveals transcriptomic difference between human tumefactive demyelinating lesion and glioma
Source: Commun Biol. 2022 Sep 9;5:941. doi: 10.1038/s42003-022-03900-0 (PMC9463163; doi:10.1038/s42003-022-03900-0)
Supplement: Supplementary file 3 — Description of Additional Supplementary Files [file 42003_2022_3900_MOESM3_ESM.pdf]

Description of Additional Supplementary Files

File name: Supplementary Data 1
Description: : Source data for figure panels 1f, 1g, 2d, 2e, 3c.

File name: Supplementary Data 2
Description: Differentially expressed genes (DEGs) that are upregulated or downregulated in cells of TDL by comparing to cells of glioma.

File name: Supplementary Data 3
Description: : Differentially expressed genes (DEGs) that are upregulated or downregulated in glial cells (C5) of TDL by comparing to glial cells (C5) of glioma.

File name: Supplementary Data 4
Description: : Differentially expressed genes (DEGs) that are upregulated or downregulated in T cells of TDL by comparing to T cells of glioma.
