# Supplementary material for: Integrative and comparative single-cell analysis reveals transcriptomic difference between human tumefactive demyelinating lesion and glioma
Source: Commun Biol. 2022 Sep 9;5:941. doi: 10.1038/s42003-022-03900-0 (PMC9463163; doi:10.1038/s42003-022-03900-0)
Supplement: Supplementary file 5 — reporting-summary [file 42003_2022_3900_MOESM5_ESM.pdf]

nature research

Corresponding author(s): Chen-Yu Ding, De-Zhi Kang, Yuan-Xiang Lin

Last updated by author(s): Aug 16, 2022

Reporting Summary

Nature Research wishes to improve the reproducibility of the work that we publish. This form provides structure for consistency and transparency in reporting. For further information on Nature Research policies, see our Editorial Policies and the Editorial Policy Checklist.

Statistics

For all statistical analyses, confirm that the following items are present in the figure legend, table legend, main text, or Methods section.

n/a	Confirmed	
☐	☒	The exact sample size (n) for each experimental group/condition, given as a discrete number and unit of measurement
☐	☒	A statement on whether measurements were taken from distinct samples or whether the same sample was measured repeatedly
☐	☒	The statistical test(s) used AND whether they are one- or two-sided<br>*Only common tests should be described solely by name; describe more complex techniques in the Methods section.*
☒	☐	A description of all covariates tested
☒	☐	A description of any assumptions or corrections, such as tests of normality and adjustment for multiple comparisons
☐	☒	A full description of the statistical parameters including central tendency (e.g. means) or other basic estimates (e.g. regression coefficient) AND variation (e.g. standard deviation) or associated estimates of uncertainty (e.g. confidence intervals)
☐	☒	For null hypothesis testing, the test statistic (e.g. F, t, r) with confidence intervals, effect sizes, degrees of freedom and P value noted<br>*Give P values as exact values whenever suitable.*
☒	☐	For Bayesian analysis, information on the choice of priors and Markov chain Monte Carlo settings
☒	☐	For hierarchical and complex designs, identification of the appropriate level for tests and full reporting of outcomes
☐	☒	Estimates of effect sizes (e.g. Cohen's d, Pearson's r), indicating how they were calculated

Our web collection on statistics for biologists contains articles on many of the points above.

Software and code

Policy information about availability of computer code

Data collection	RNA sequencing libraries were constructed and loaded on an Illumina NovaSeq 6000 sequencer for sequencing.
Data analysis	Raw gene expression matrices of RNA-seq data were loaded into the statistical computing environment R (4.0.4) and processed according to the Seurat R package. The processed data from RNA-seq were analysed using GraphPad Prism 8 for statistical analysis.

For manuscripts utilizing custom algorithms or software that are central to the research but not yet described in published literature, software must be made available to editors and reviewers. We strongly encourage code deposition in a community repository (e.g. GitHub). See the Nature Research guidelines for submitting code & software for further information.

Data

Policy information about availability of data

All manuscripts must include a data availability statement. This statement should provide the following information, where applicable:

- Accession codes, unique identifiers, or web links for publicly available datasets
- A list of figures that have associated raw data
- A description of any restrictions on data availability

The original transcriptomic data generated during this study are publicly available in Gene Expression Omnibus (accession ID: GSE202096).

Field-specific reporting

Please select the one below that is the best fit for your research. If you are not sure, read the appropriate sections before making your selection.

☒ Life sciences ☐ Behavioural & social sciences ☐ Ecological, evolutionary & environmental sciences

For a reference copy of the document with all sections, see nature.com/documents/nr-reporting-summary-flat.pdf

Life sciences study design

All studies must disclose on these points even when the disclosure is negative.

Sample size	No statistical methods were used to predetermine sample size.
Data exclusions	Three quality criteria were applied to filter the matrices for excluding low-quality cells, including number of expressed genes (>200), number of detected transcripts (<99%), and proportion of mitochondrial genes (≤25%).
Replication	The case (TDL) is rare which is hard to repeat.
Randomization	Randomization is not relevant to this work as the two disease is different.
Blinding	No blinding was used as the results were mainly derived from unsupervised analysis.

Reporting for specific materials, systems and methods

We require information from authors about some types of materials, experimental systems and methods used in many studies. Here, indicate whether each material, system or method listed is relevant to your study. If you are not sure if a list item applies to your research, read the appropriate section before selecting a response.

Materials & experimental systems

n/a	Involved in the study	
☒	☐	Antibodies
☒	☐	Eukaryotic cell lines
☒	☐	Palaeontology and archaeology
☒	☐	Animals and other organisms
☐	☒	Human research participants
☒	☐	Clinical data
☒	☐	Dual use research of concern

Methods

n/a	Involved in the study	
☒	☐	ChIP-seq
☒	☐	Flow cytometry
☒	☐	MRI-based neuroimaging

Human research participants

Policy information about studies involving human research participants

Population characteristics	The two patients in our study were diagnosed as WHO III grade glioma (male, 28 years old) or TDL (female, 61 years old).
Recruitment	Patients who were diagnosed as WHO III grade glioma or TDL and underwent surgery at The First Affiliated Hospital of Fujian Medical University were included in this study. The high heterogeneity and little sample are the potential bias and. The different sex and age in patients may impact results.
Ethics oversight	The Ethics Committee of The First Affiliated Hospital, Fujian Medical University.

Note that full information on the approval of the study protocol must also be provided in the manuscript.
